# Long-acting cabotegravir pharmacokinetics with and without oral lead-in for HIV PrEP

**DOI:** 10.1128/aac.01475-23

**Published:** 2024-05-06

**Authors:** Kelong Han, Parul Patel, Scott McCallister, Alex R. Rinehart, Yash Gandhi, William Spreen, Raphael J. Landovitz, Sinead Delany-Moretlwe, Mark A. Marzinke, Todd McKeon, Piotr Budnik, Jean van Wyk, Susan L. Ford

**Affiliations:** 1 GSK, Collegeville, Pennsylvania, USA; 2 ViiV Healthcare, Durham, North Carolina, USA; 3 ViiV Healthcare, Branford, Connecticut, USA; 4 Center for Clinical AIDS Research and Education, David Geffen School of Medicine, University of California, Los Angeles, California, USA; 5 Wits RHI, University of the Witwatersrand, Johannesburg, South Africa; 6 Johns Hopkins University, Baltimore, Maryland, USA; 7 ViiV Healthcare, Brentford, United Kingdom; 8 GSK, Durham, North Carolina, USA; University of Pittsburgh, Pittsburgh, Pennsylvania, USA

**Keywords:** cabotegravir, oral lead-in, pharmacokinetics, HIV, pre-exposure prophylaxis

## Abstract

Long-acting cabotegravir is approved for pre-exposure prophylaxis and combination HIV treatment, both initiated with optional short-term oral lead-in (OLI). We evaluated the impact of OLI on long-acting cabotegravir pharmacokinetics. Cabotegravir plasma concentrations were compared between HIV-positive participants initiating injections with (*n* = 278) or without (*n* = 110) OLI in phase III treatment study FLAIR and in HIV-negative participants using OLI (*n* = 263) in pivotal pre-exposure prophylaxis studies HPTN 083 and HPTN 084. Cabotegravir pharmacokinetic profiles were simulated in three populations (assigned-male-at-birth, 50%-assigned-female-at-birth, and assigned-female-at-birth) under three scenarios: first injection given (A) 1 or (B) 3 days after final OLI dose (OLI-injection gap) or (C) without OLI. The PK objective was 80% of participants achieving 4× *in vitro* protein-adjusted 90% maximal inhibitory concentration (PA-IC_90_) and 50% achieving 8× PA-IC_90_. Observed trough concentrations (*C*_τ_) were similar with and without OLI (*P* > 0.3). With a 3-day OLI-injection gap, simulated pre-injection *C*_τ_ remained above PK objective. Approximately 1–2 weeks after the first injection, simulated PK profiles became nearly identical among all scenarios. Without OLI, it was predicted that 80% of participants achieve 4× PA-IC_90_ in 1.2, 1.8, and 2.8 days after the first injection in each population, respectively, and 50% achieve 8× PA-IC_90_ in 1.4, 2.1, and 3.8 days, respectively. Observed long-acting cabotegravir exposure was similar with or without OLI, supporting optional OLI use. Cabotegravir exposure was predicted to remain above PK objective for OLI-injection gaps of ≤3 days and rapidly achieve PK objective after first injection without OLI. Findings are consistent between assigned-male-at-birth and assigned-female-at-birth populations.

**CLINICAL TRIALS:**

This study is registered with ClinicalTrials.gov as NCT02720094.

## INTRODUCTION

Introduction of antiretroviral agents that are highly effective in reducing the risk of HIV-1 acquisition as pre-exposure prophylaxis (PrEP) has been a significant advance toward ending the HIV/AIDS epidemic (1–5). Oral PrEP with tenofovir disoproxil fumarate/emtricitabine (TDF/FTC) or tenofovir alafenamide fumarate/FTC is approved for certain populations, but efficacy is adherence dependent (6, 7), which may be a challenge for some individuals. In addition, the burden and anxiety of daily oral dosing may also impact quality of life and may lead to inadvertent disclosure to others and negative stigma (8, 9). The availability of long-acting (LA) forms of PrEP may overcome challenges associated with daily oral dosing (10, 11).

Cabotegravir (CAB), an integrase strand transfer inhibitor, is the first single-agent LA injectable approved for HIV PrEP that is administered intramuscularly by a healthcare provider once every 2 months (Q2M), with the first 2 injections given 1 month apart (3, 4, 12, 13). Results from two global, pivotal, randomized, double-blind, double-dummy PrEP studies in 4,566 (*n* = 2,282 randomized to active CAB LA, *n* = 2,284 to active TDF/FTC) men who have sex with men and transgender women (HPTN 083, ClinicalTrials.gov NCT02720094) and 3,224 (*n* = 1,614 to active CAB LA, *n* = 1,610 to active TDF/FTC) cisgender women (HPTN 084, ClinicalTrials.gov NCT03164564) demonstrated CAB LA Q2M was well-tolerated and superior to daily oral TDF/FTC in preventing HIV acquisition (3, 4). Participants in the CAB LA group had 66% (HPTN 083) and 88% (HPTN 084) lower risk of HIV acquisition as compared with participants randomized to oral TDF/FTC. Previously, CAB LA, in combination with rilpivirine (RPV) LA, administered Q2M, demonstrated non-inferior efficacy to the monthly regimen for the maintenance of HIV-1 virologic suppression, leading to its subsequent approval and guideline recommendation for HIV-1 treatment (14–20).

Cabotegravir is available as an LA injectable and as an oral tablet (30 mg once daily) that is used to cover planned interruptions in injection dosing (oral bridging therapy) or to assess individual tolerability to CAB during short-term oral lead-in (OLI) therapy (1). In the phase III FLAIR (201584, ClinicalTrials.gov NCT02938520), ATLAS (201585, ClinicalTrials.gov NCT02951052), and ATLAS-2M (207966, ClinicalTrials.gov NCT03299049) HIV treatment studies, as well as the HPTN 083 and 084 PrEP studies, participants initiated therapy with CAB OLI for up to 5 weeks, and the first LA injection was administered within 24 hours after the final OLI dose (3, 4, 15–17). Subsequent to demonstrating comparable efficacy, safety, and pharmacokinetics (PK) with and without OLI during the extension phase of FLAIR and the extensive and favorable safety profile across treatment and PrEP studies, OLI has been approved for optional use and is no longer required (19).

Cabotegravir reaches maximum plasma concentrations approximately 1 week post CAB LA injections (21). Oral CAB has a mean elimination half-life of 31–45 hours. Therefore, the ongoing contribution of OLI to systemic exposure is expected to be limited to the first 1 to 2 weeks following the final oral dose, with no impact on trough concentration (*C*_τ_) 1 month after the first LA injection (22–24). Omitting OLI or allowing a longer gap between the final OLI dose and the first CAB LA injection in PrEP could provide additional convenience for the participants and accommodate appointment scheduling.

Our objective was to evaluate CAB exposure and the time to reach CAB plasma concentrations associated with significant antiviral activity (above the *in vitro* protein-adjusted 90% maximal inhibitory concentration [PA-IC_90_]) using both observed and simulated PK data in various OLI dosing scenarios. The PK objective in this analysis was to achieve target plasma CAB concentrations >1× PA-IC_90_ in 95% of participants, >4× PA-IC_90_ in 80% of participants, and >8× PA-IC_90_ in 50% of participants, based on concentrations affording protection in non-human primate challenge studies (25–27).

## RESULTS

### Participants

Demographics of participants with PK data (defined as at least 1 of the 3 plasma concentrations of interest: before the first and second injection or 1 week after the first injection) is provided in Table 1. In FLAIR, PK data were collected from 110 participants without OLI and 278 participants with OLI. In HPTN 083, all participants with PK data (*n* = 114) were assigned male at birth. In HPTN 084, all participants with PK data (*n* = 149) were assigned female at birth.

**TABLE 1:** Baseline characteristics by study^*a*^

Parameter	Participants without OLI	Participants with OLI		
	FLAIR (*n* = 110)	FLAIR (*n* = 278)	HPTN 083 (*n* = 114)	HPTN 084 (*n* = 149)
Age, yr	33 (18–68)	34 (19–68)	24 (18–60)	25 (18–43)
Female at birth, *n* (%)	23 (21)	61 (22)	0	149 (100)
Race, *n* (%)				
White	76 (69)	211 (76)	14 (12)	0
Black or African American	23 (21)	47 (17)	20 (18)	149 (100)
Asian	8 (7)	12 (4)	53 (46)	0
American Indian or Alaska Native	3 (3)	3 (1)	24 (21)	0
Other	0	5 (2)	2 (2)	0
Unknown	0	0	1 (1)	0
Body weight, kg	75 (45.9–128.1)	74 (46–125.6)	66.6 (42.5–161.6)	67 (41–127)
BMI, kg/m^2^	24.4 (18–47.4)	24.1 (17.3–44.9)	23 (15–53)	27 (17–53)

^*a*^ Age, body weight, and BMI are displayed as median (range); BMI, body mass index; *n*, number of participants with PK data; OLI, oral lead-in.

### Comparison of observed CAB plasma concentrations with and without OLI

**Fig 1:**
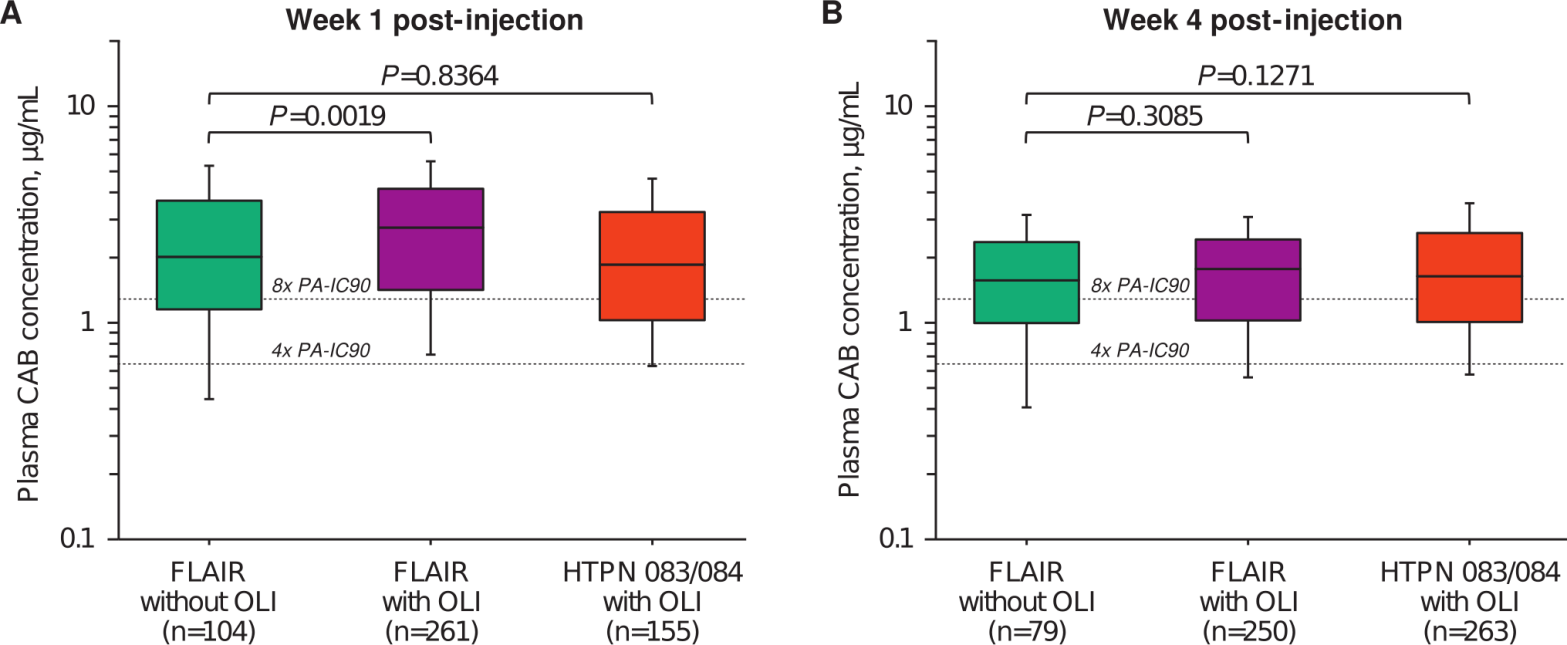
Cabotegravir plasma concentrations by study (**A**) 1 week after the first injection and (**B**) 4 weeks after the first injection. CAB, cabotegravir; *n*, number of participants; OLI, oral lead-in.

Cabotegravir plasma concentrations before the first 600 mg intramuscular (IM) CAB LA injection at 1 week and 4 weeks after the first injection are summarized in Table 2 and Fig. 1.

**TABLE 2:** Observed cabotegravir plasma concentrations with or without OLI by study^*b*^

PK time point	Cabotegravir plasma concentration geometric mean [95% CI] (5th and 95th percentiles), μg/mL				
	Phase III treatment study FLAIR (male and female at birth)		Pivotal PrEP studies		
			HPTN 083 (male at birth)	HPTN 084 (female at birth)	Combined HPTN 083 and HPTN 084
	Without OLI	With OLI	With OLI	With OLI	With OLI
Before the first LA injection	0	5.22 [4.88, 5.57] (2.42, 9.91) *n* = 243	4.29 [2.90, 6.35] (0.025, 12.0) *n* = 58^*a*^	3.08 [2.30, 4.11] (0.03, 12.2) *n* = 149^*a*^	3.47 [2.78, 4.33] (0.03, 12.0) *n* = 207^*a*^
1 week after the first LA injection (~*T*_max_)	1.89 [1.61, 2.22] (0.44, 5.69) *n* = 104	2.52 [2.31, 2.75] (0.71, 7.52) *n* = 261	2.90 [2.30, 3.65] (0.55, 10.6) *n* = 58	1.51 [1.33, 1.72] (0.61, 3.94) *n* = 97	1.93 [1.70, 2.18] (0.63, 8.27) *n* = 155
4 weeks after the first LA injection (*C*_τ_ before injection 2)	1.43 [1.24, 1.66] (0.41, 3.89) *n* = 79	1.56 [1.45, 1.68] (0.56, 3.61) *n* = 250	1.86 [1.63, 2.12] (0.43, 4.73) *n* = 114	1.48 [1.35, 1.62] (0.65, 4.40) *n* = 149	1.63 [1.51, 1.76] (0.58, 4.56) *n* = 263

^*a*^ Number of samples below the lower limit of quantification: 3 for HPTN 083 and 6 for HPTN 084.

^*b*^ *C*_τ_, observed trough concentration; LA, long-acting; *n*, number of participants; OLI, oral lead-in; PK, pharmacokinetic; PrEP, pre-exposure prophylaxis; *T*_max_, time to maximum plasma concentration.

In FLAIR, the geometric mean CAB plasma concentration before injection initiation was 5.22 µg/mL (*n* = 243) in participants who received OLI and was non-quantifiable in participants who declined OLI (*n* = 110) as expected. One week after the first LA injection, CAB plasma concentrations were higher in participants with OLI than participants without OLI (geometric mean 2.52 µg/mL vs 1.89 µg/mL, *P* = 0.0019). However, 4 weeks after the first LA injection, CAB plasma concentrations were similar in participants with and without OLI (geometric mean 1.56 µg/mL vs 1.43 µg/mL, *P* = 0.3085).

In the HPTN 083 and 084 PrEP studies, geometric mean CAB plasma concentrations before CAB LA injection initiation were 4.29 µg/mL (*n* = 58) and 3.08 µg/mL (*n* = 149), respectively. Geometric mean CAB plasma concentrations at 1 week and 4 weeks after the first LA injection in HPTN 083 were 2.90 µg/mL and 1.86 µg/mL, respectively, and were 1.51 µg/mL and 1.48 µg/mL in HPTN 084, respectively. Both 1 week and 4 week post-injection concentrations of the combined HPTN 083 and HPTN 084 PK data set were similar to participants in FLAIR who received injections without OLI (*P* = 0.8364 and *P* = 0.1271, respectively).

### Comparison of simulated CAB plasma concentrations

**Fig 2:**
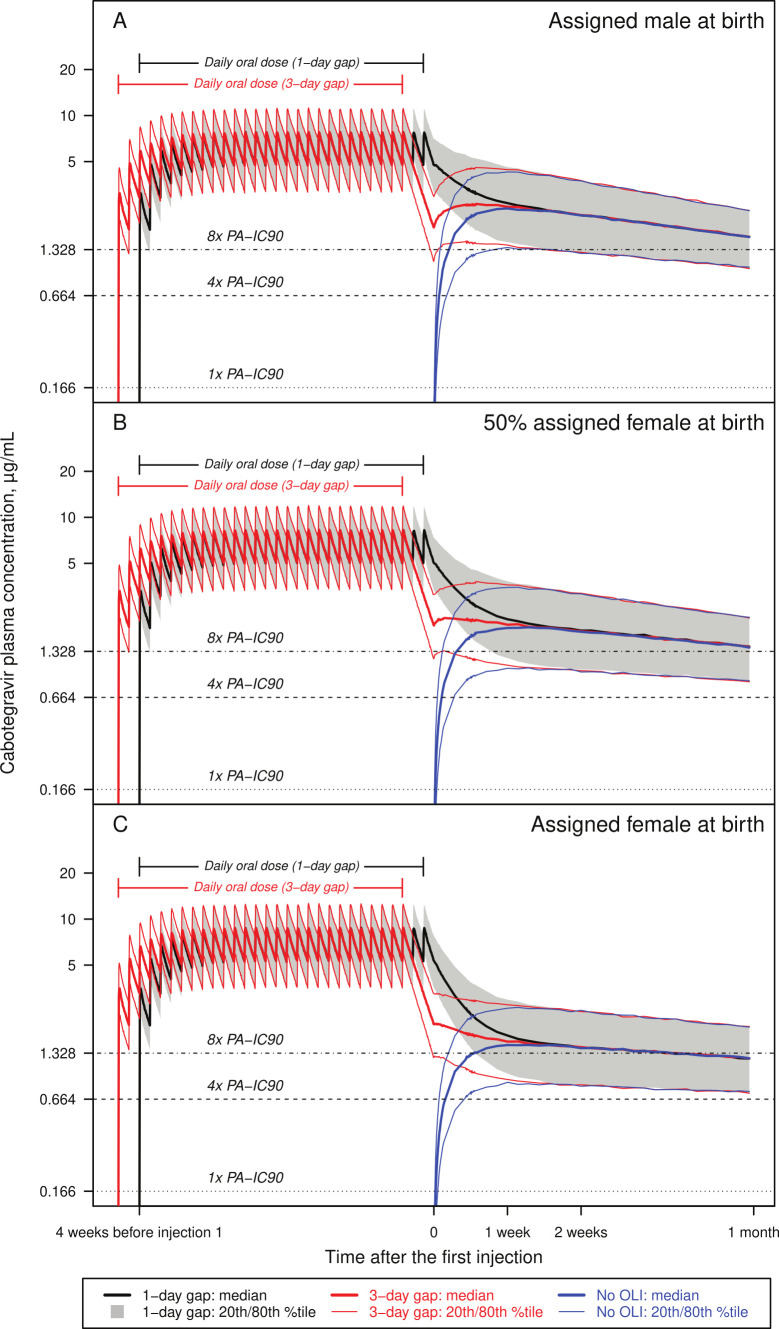
Simulated cabotegravir concentration-time profiles when the first injection is given with a 1-day or 3-day gap after the final OLI dose or without OLI in a population (**A**) assigned male at birth, (**B**) 50% assigned female at birth, and (**C**) assigned female at birth. The first injection is administered at time 0. %tile, percentile; *C*_τ_, trough concentration; OLI, oral lead-in; PA-IC90, *in vitro* protein-adjusted 90% maximal inhibitory concentration.

Within each of the three populations (0%, 50%, and 100% assigned female at birth), simulated CAB PK profiles were compared among the three scenarios (Fig. 2): (A) first LA injection given 1 day after the final oral dose (OLI-injection gap), (B) first LA injection given 3 days after the final oral dose, and (C) first LA injection given without OLI.

#### CAB PK before the first injection

Although a 1-day OLI-injection gap was predicted to lead to higher *C*_τ_ before the first injection (*C*_τ_−0) than a 3-day gap (time 0 in Fig. 2), *C*_τ_−0 was predicted to stay above the PK objective in both scenarios (Table 3). Over 99.9% of participants across all three populations were predicted to maintain *C*_τ_−0 above PA-IC_90_ with a 1-day or 3-day gap, both exceeding the PK objective of 95% above PA-IC_90_. Approximately 95% of participants across all three populations were predicted to maintain *C*_τ_−0 above 4× PA-IC_90_ with a 3-day gap, vs 99.9% with a 1-day gap, both exceeding the PK objective of 80% above 4× PA-IC_90_. Approximately 70%–76% of participants across all three populations were predicted to maintain *C*_τ_−0 above 8× PA-IC_90_ with a 3-day gap, vs 99% with a 1-day gap, both exceeding the PK objective of 50% above 8× PA-IC_90_.

**TABLE 3:** Predicted proportion of participants receiving OLI and maintaining 4× and 8× PA-IC_90_ before the first injection^*a*^

Population	Gap between the final oral dose and the first injection	Predicted % of participants with *C*_τ_ >4× PA-IC_90_ (expectation: >80%)	Predicted % of participants with *C*_τ_ >8× PA-IC_90_ (expectation: >50%)
Assigned male at birth	1 day	99.9	98.8
	3 days	94.7	70.6
50% assigned female at birth	1 day	99.9	99.1
	3 days	95.4	73.1
Assigned female at birth	1 day	99.9	99.1
	3 days	95.9	76.1

^*a*^ *C*_τ_, trough concentration; OLI, oral lead-in; PA-IC_90_, *in vitro* protein-adjusted 90% maximal inhibitory concentration. Predicted percentage of participants with *C*_τ_ >PA-IC_90_ is not displayed because all values are >99.9%, exceeding 95%. Predicted percentage for scenario C (no OLI) is not displayed because all values are zero.

#### CAB PK after the first injection

**Fig 3:**
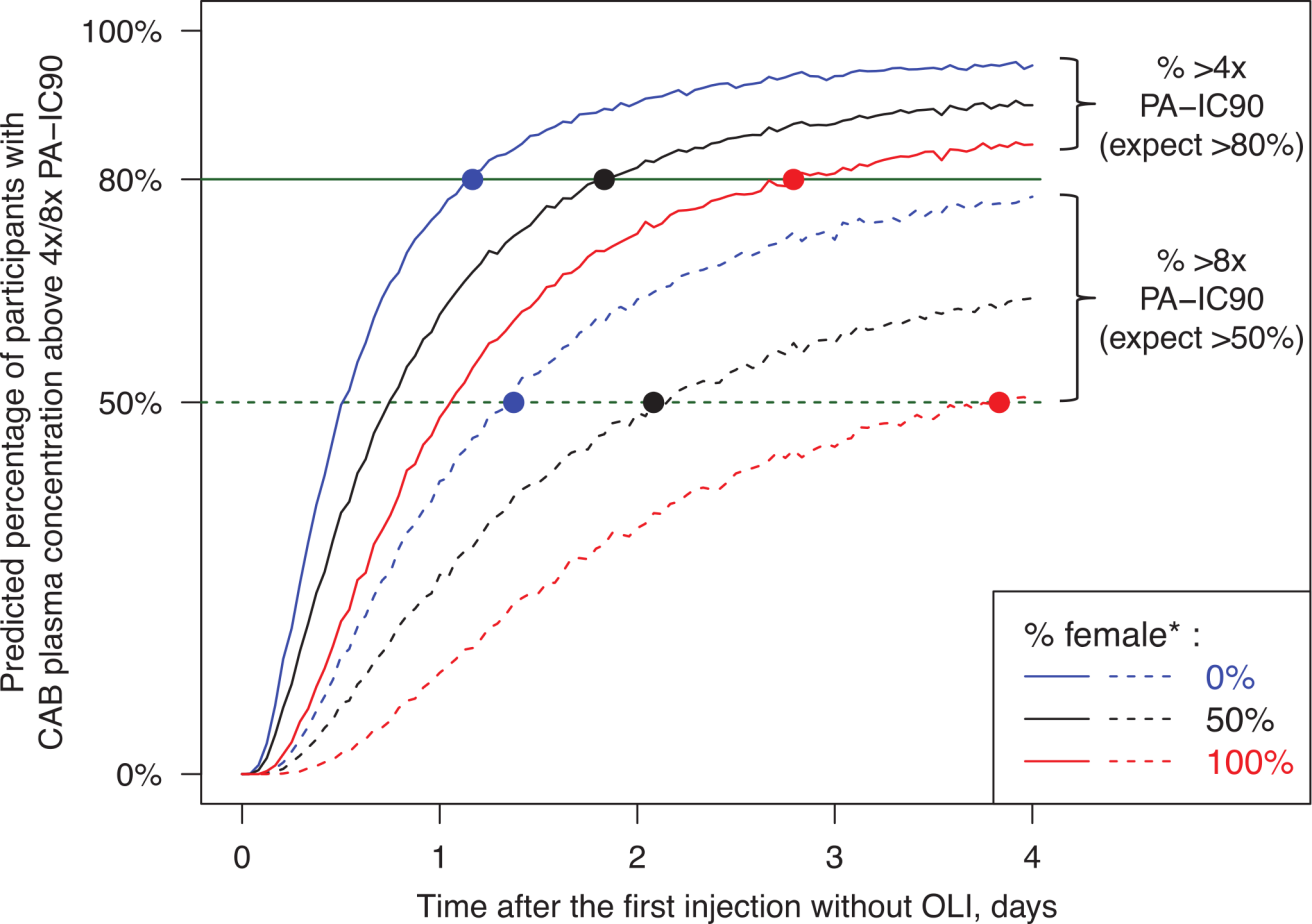
Predicted percentage of participants achieving 4× PA-IC_90_ (solid lines) and 8× PA-IC_90_ (dashed lines) over time after the first injection without OLI in a population assigned male at birth (blue), 50% assigned female at birth (black), and assigned female at birth (red). Solid cycles represent the predicted time to achieve the PK objective of 80% above 4× PA-IC_90_ and 50% above 8× PA-IC_90_. CAB, cabotegravir; OLI, oral lead-in; PA-IC90, *in vitro* protein-adjusted 90% maximal inhibitory concentration; PK, pharmacokinetic. *Assigned female at birth.

Approximately 1–2 weeks after the first injection, the simulated CAB PK profiles including *C*_τ_ (time of 1 month in Fig. 2) became nearly identical among the three scenarios with or without OLI (Fig. 2). With OLI (scenarios A and B), nearly 100% of participants were predicted to achieve 1×, 4×, and 8× PA-IC_90_ immediately after the first injection, likely caused by ongoing contribution from the oral dosing. Without OLI (scenario C, Table 4; Fig. 3), it was predicted to take:

- 10, 15, and 19 hours (0.4, 0.6, and 0.8 days) to achieve the PK objective of 95% of participants above PA-IC_90_ after the first injection in the three populations (0%, 50%, and 100% assigned female at birth), respectively
- 28, 44, and 67 hours (1.2, 1.8, and 2.8 days) to achieve the PK objective of 80% of participants above 4× PA-IC_90_ after the first injection in the three populations, respectively
- 33, 50, and 92 hours (1.4, 2.1, and 3.8 days) to achieve the PK objective of 50% of participants above 8× PA-IC_90_ after the first injection in the three populations, respectively

**TABLE 4:** Predicted time to achieve the PK objective after the first injection without OLI^*a*^

Population	>1× PA-IC_90_ in 95% of participants	>4× PA-IC_90_ in 80% of participants	>8× PA-IC_90_ in 50% of participants
Assigned male at birth	10 h (0.4 d)	28 h (1.2 d)	33 h (1.4 d)
50% assigned female at birth	15 h (0.6 d)	44 h (1.8 d)	50 h (2.1 d)
Assigned female at birth	19 h (0.8 d)	67 h (2.8 d)	92 h (3.8 d)

^*a*^ OLI, oral lead-in; PA-IC_90_, in vitro protein-adjusted 90% maximal inhibitory concentration.

## DISCUSSION

In this analysis, we compared observed CAB plasma concentrations within the first 4 weeks following an initial 600 mg IM injection among participants in various pivotal studies who received the first CAB LA injection with or without OLI. In addition, model-based simulations were performed to generate CAB plasma concentrations after initiating CAB LA IM Q2M in three scenarios: first injection given (A) 1 or (B) 3 days after final OLI dose or (C) without OLI. Results from both observed PK and simulated PK were consistent, supporting the optional use of OLI when initiating CAB LA injections in conjunction with observed efficacy and safety data across the CAB development program. These results were used as the basis for approval of optional OLI in both treatment and PrEP indications and allowance of up to a 3-day gap between final OLI dose and first CAB LA PrEP injection in product labeling.

Oral CAB has a half-life of approximately 40 hours, which contributes to the plasma PK profile for 1–2 weeks (6 half-lives) following discontinuation of oral dosing (22–24). For this reason, observed CAB plasma concentrations were higher (*P* = 0.0019) at 1 week post-injection in participants with OLI than participants without OLI in FLAIR (Fig. 1) (22–24), and the simulated CAB PK profiles became nearly identical with or without OLI approximately 1–2 weeks after the first injection (Fig. 2). By 4 weeks post-injection, there was no ongoing contribution from the final oral CAB dose, as reflected by the lack of difference in CAB plasma concentration with or without OLI in participants from FLAIR (*P* = 0.3085) and HPTN 083 and 084 studies (*P* = 0.1271).

The absorption rate for CAB LA is approximately 50% lower in females assigned at birth than males assigned at birth (23), leading to lower maximum plasma concentrations and *C*_τ_ in females than males after the first injection, as evidenced by the lower concentrations after the first injection in HPTN 084 than HPTN 083 (Table 2). Therefore, data from HPTN 083 and HPTN 084 were combined for purposes of comparison with the mixed-sex population in FLAIR who received OLI. For the same reason, simulations were performed separately in the three populations (0%, 50%, and 100% assigned female at birth). Results of simulated PK are consistent between persons assigned male and female at birth:

- Before the first injection with a 3-day OLI-injection gap: 94.7% male vs 95.9% female participants stayed >4× PA-IC_90_, and 70.6% male vs 76.1% female achieved >8× PA-IC_90_
- After the first injection without OLI, although it would take approximately twice as long for female participants to achieve the PK objective as male participants, the PK objective would be achieved rapidly for both male (≤1.4 days) and female (≤3.8 days) participants. The difference in the predicted time to achieve the PK objective between male and female assigned at birth ranged from 0.4 days to 2.4 days. It would take (male vs female assigned at birth):
  - 10 vs 19 hours (0.4 vs 0.8 days) to reach 95% participants > PA-IC_90_
  - 28 vs 67 hours (1.2 vs 2.8 days) to reach 80% participants >4× PA-IC_90_
  - 33 vs 92 hours (1.4 vs 3.8 days) to reach 50% participants >8× PA-IC_90_

Therefore, despite slower CAB LA absorption in persons assigned female at birth, a 3-day gap or optional OLI are not expected to lead to increased risk of HIV seroconversion.

Simulated CAB plasma concentrations were generated using a robust population pharmacokinetic (PPK) model previously built based on a total of 23,926 CAB plasma concentrations collected from 1,647 HIV-negative and HIV-positive adult participants (23), and CAB PK profiles following a Q2M regimen were simulated with 5,000 virtual participants. As expected, with OLI, 1×, 4×, and 8× PA-IC_90_ were predicted to be achieved almost immediately in nearly 100% of participants due to residual oral exposure. Without OLI, the PK objective was predicted to be rapidly achieved after the first CAB LA injection (Table 4; Fig. 3). Although intensive PK samples were not collected within 1 week post-injection in FLAIR, CAB exposure within 1 week post-injection is anticipated to be similar as the simulation, especially considering that no difference in efficacy, safety, and PK profiles in suppressed participants switching to CAB+RPV LA with or without OLI was observed in FLAIR (28). Altogether, and with the robustness of the PPK model allowing for the use of model-predicted results, this PK analysis indicates that initiating CAB LA without OLI does not result in a notable delay in reaching 1×, 4×, and 8× PA-IC_90_, supporting the optional use of OLI.

The PPK model showed a correlation between absorption rate for CAB LA and body mass index (BMI). Body mass index has no impact on CAB PK after oral dosing, and the impact of BMI on CAB LA PK is the same with or without OLI. Therefore, the conclusion that CAB LA exposure was similar with or without OLI is applicable to all BMI categories, supporting optional OLI use regardless of BMI category.

In HPTN 083 and 084, CAB LA demonstrated superiority over TDF/FTC to prevent HIV acquisition (3, 4, 29). Overall, participants in the CAB LA group had a 66% (HPTN 083) and 88% (HPTN 084) lower risk of acquiring HIV than participants in the TDF/FTC group. Incidence of HIV acquisition after CAB LA exposure was extremely low (4, 29). Due to the high level of efficiency at preventing HIV acquisition and the multifactorial aspect for risk of HIV acquisition, the exact time from initiation of CAB LA for HIV-1 PrEP to maximal protection against HIV-1 infection is unknown. However, based on simulations, CAB plasma concentrations considered having significant antiviral activity are reached and maintained within 7 days after the first injection without OLI.

For participants opting for CAB PrEP with OLI, CAB plasma concentrations were predicted to remain above the PK objective for OLI-injection gaps of ≤3 days, enabling scheduling flexibility in PrEP. For a 4-day gap in PrEP, CAB plasma concentration before the first injection was predicted to be >8× PA-IC_90_ in 41.2% (male) to 47.6% (female) of participants, below 50%, although time to achieve the PK objective after the first injection was predicted to be similar to a 3-day gap. For participants opting for CAB PrEP without OLI, the PK objective is predicted to be achieved within 1–4 days after the first injection.

For people living with HIV opting to receive CAB LA for treatment with OLI, although a gap of ≤3 days is expected to lead to similar CAB plasma concentrations as PrEP, it is recommended that initiation injections be administered on the final day of OLI to maintain consistent antiviral treatment dosing and ongoing suppression of HIV infection. For people living with HIV receiving CAB LA for treatment without OLI, prior active antiretroviral therapy should be administered up to the date of initiation injections to maintain continuity of antiretroviral therapy.

There were limitations to this analysis. Cabotegravir plasma concentrations in HIV-negative individuals who do not use OLI or have up to a 3-day OLI-injection gap have been derived only from simulations and not from clinical studies given the design of the HPTN 083 and 084 studies, and adherence to oral dosing was assessed by pill count at 2 and 4 weeks after the first oral dose in all studies. However, due to the robustness of the CAB PPK model, findings in this analysis remain valid. In addition, data in participants initiating CAB LA without OLI or after a longer OLI-injection gap are being actively collected in the HPTN 083 and 084 studies. The other limitation is that tissue samples were not collected in the FLAIR and HPTN 083 and 084 studies; therefore, tissue concentrations, inoculum size, or potential mucosal or vaginal membrane disruptions could not be evaluated in this analysis. However, a recent study demonstrated a strong correlation (adjusted *R*^2^ >0.75) between CAB plasma concentrations and time-matched cervical, vaginal, and rectal tissue CAB concentrations, with similar distribution of CAB into tissues and plasma from oral dosing and IM injection (30). The plasma and tissue concentration-vs-time profiles were parallel, indicating that the distribution of CAB into tissues causes no delay and therefore should have no impact on the results of time to achieve the PK objective demonstrated in this analysis.

In conclusion, CAB exposure following LA injections was similar with or without OLI, supporting that the OLI be optional. Cabotegravir plasma concentrations were predicted to remain above the PK objective for gaps of ≤3 days between final oral dose and first injection. Without an OLI, the PK objective was predicted to be rapidly achieved after the first injection. These findings and recommendations are consistent between persons assigned male at birth and those assigned female at birth. These results support CAB LA as a highly flexible and efficacious alternative to daily oral PrEP for individuals at risk of HIV acquisition.

## MATERIALS AND METHODS

### Study design and population

FLAIR (17) was a phase III randomized study evaluating CAB+RPV LA IM monthly injection vs daily oral comparator therapy of dolutegravir/abacavir/lamivudine or dolutegravir +2 alternative nucleoside/nucleotide reverse transcriptase inhibitors in people living with HIV. At the start of the Extension Phase, participants in the oral comparator group who successfully completed Week 100 could either switch to CAB+RPV LA or be withdrawn from the study. Participants choosing to receive CAB+RPV LA in the Extension Phase were given the option to transition to CAB+RPV LA with or without OLI. Cabotegravir plasma concentrations were measured in all participants.

In HPTN 083 (3) and 084 (4), HIV-negative cisgender men and transgender women aged ≥18 years (HPTN 083) and cisgender women aged 18–45 years (HPTN 084) were randomized 1:1 to receive CAB LA 600 mg IM Q2M regimen or oral TDF/FTC once daily for PrEP. Participants in the CAB LA group received OLI for 5 weeks, which was stopped approximately 1 day before the initiation injection of IM CAB LA 600 mg at Week 5 followed by Q2M maintenance injections of IM CAB LA 600 mg starting at Week 9. Cabotegravir plasma concentrations were measured in a portion of the participants.

Adherence to oral dosing was assessed by pill count at 2 and 4 weeks after the first oral dose in all studies. All studies were conducted with the approval of center ethics committees or institutional review board and in accordance with principles outlined in the Declaration of Helsinki. Written informed consent was provided by all participant before study initiation.

### Comparison of observed CAB plasma concentrations with and without OLI

Cabotegravir plasma concentrations observed at 1 week and 4 weeks after the first injection in participants initiating CAB LA without OLI from FLAIR were (i) compared with all participants from FLAIR who received OLI, including all participants from the CAB+RPV LA arm and participants from the oral comparator arm who chose OLI and (ii) compared with the combined data from the HPTN 083 and 084 PrEP studies in HIV-negative participants initiating CAB LA with OLI given that the first LA dose was the same in FLAIR and HPTN 083 and 084. Non-quantifiable CAB plasma concentrations were imputed as the lower limit of quantification of 0.025 µg/mL. The difference in CAB plasma concentrations was tested using an unpaired 2-tailed *t* test. A *P* value of < 0.05 was considered to be statistically significant.

### PK objective

An exposure-response relationship between CAB plasma PK parameters and efficacy endpoints (virologic response for treatment and seroconversion for PrEP) is difficult to establish given the low rate of virologic failure for treatment and the low rate of seroconversion for PrEP in pivotal studies (3, 4, 28). In non-human primate studies, CAB plasma concentrations ≥1× PA-IC_90_ conferred 97% protection in male macaques undergoing repeat rectal challenge with simian-human immunodeficiency virus (25). Cabotegravir plasma concentrations >4× PA-IC_90_ afforded 98% protection in female rhesus macaques undergoing repeat intravaginal challenge with simian immunodeficiency virus, and concentrations >1× PA-IC_90_ provided 97% protection in female rhesus macaques undergoing repeated intravaginal challenge with high-dose simian-human immunodeficiency virus while receiving medroxyprogesterone (26, 27). Based on the CAB concentrations that afforded protection in these non-human primate studies, the CAB IM Q2M PrEP regimen was designed to achieve CAB *C*_τ_ of >1×, >4×, and >8× PA-IC_90_ in 95%, 80%, and 50% of participants, respectively (31). The PK objective for PrEP used in this analysis were 95% of participants achieving >1× PA-IC_90_, 80% achieving >4× PA-IC_90_, and 50% achieving >8× PA-IC_90_.

### Population PK (PPK) Simulations

Long-acting cabotegravir PK profiles were simulated in 5,000 virtual subjects following the CAB LA IM Q2M regimen using a previously published PPK model (23). Covariates of sex, body weight, and body mass index were simulated using the relationships between these covariates that were established using the PPK model-building data set. Individual PK parameters of the virtual subjects were calculated using subject-specific covariates and using the population parameter estimates, subject-specific inter-individual errors (ETA) sampled from the distributions that are decided by the estimated variance-covariate matrix of inter-individual variability from the final PPK model. Residual variability was included in the simulations.

Simulations were constructed for three scenarios: (A) first LA injection given 1 day (24 hours) after the final oral dose, (B) first LA injection given 3 days (72 hours) after the final oral dose, and (C) first LA injection given without OLI. Simulations were constructed for three populations: 0%, 50%, and 100% assigned female at birth. Time and proportion of participants to reach 1× (0.166 µg/mL), 4× (0.664 µg/mL), or 8× (1.328 µg/mL) PA-IC_90_ after the first LA injection were calculated and compared among the three scenarios and three populations. All analyses were performed using R (version 3.6.3).

## Data Availability

The data that support the findings of this analysis are available from GSK upon request and approval from https://www.clinicalstudydatarequest.com/.
